# Outbreak of OXA-48-producing Enterobacterales in a haematological ward associated with an uncommon environmental reservoir, France, 2016 to 2019

**DOI:** 10.2807/1560-7917.ES.2021.26.21.2000118

**Published:** 2021-05-27

**Authors:** Sarah Jolivet, Jeanne Couturier, Xavier Vuillemin, Cyril Gouot, Didier Nesa, Marine Adam, Eolia Brissot, Mohamad Mohty, Rémy A Bonnin, Laurent Dortet, Frédéric Barbut

**Affiliations:** 1 Unité d’Hygiène et de Lutte contre les Infections Nosocomiales, Hôpital Saint-Antoine, Assistance Publique-Hôpitaux de Paris, Paris, France; 2 IAME, UMR 1137, INSERM, Université de Paris, Paris, France; 3 Laboratoire de Microbiologie de l’Environnement, Hôpital Saint-Antoine, Assistance Publique-Hôpitaux de Paris, Paris, France; 4 Unité INSERM S-1139, Université de Paris, Faculté de Pharmacie, Paris, France; 5 Service d'Hématologie clinique et Thérapie cellulaire, Hôpital Saint-Antoine, Assistance Publique-Hôpitaux de Paris, Sorbonne University, INSERM UMRs 938, Paris, France; 6 Unité EA7361 “Structure, dynamic, function and expression of broad spectrum β-lactamases”, Université Paris Sud, Université Paris Saclay, LabEx Lermit, Faculty of Medicine, Le Kremlin-Bicêtre, France; 7 Bacteriology-Hygiene unit, Assistance Publique-Hôpitaux de Paris, Bicêtre Hospital, Le Kremlin-Bicêtre, France; 8 Associated French National Reference Center for Antibiotic Resistance: Carbapenemase-producing Enterobacteriaceae, Le Kremlin-Bicêtre, France

**Keywords:** outbreak, carbapenemase-producing Enterobacterales, Citrobacter freundii, reservoir, toilet

## Abstract

The hospital water environment, including the wastewater drainage system, is increasingly reported as a potential reservoir for carbapenemase-producing Enterobacterales (CPE). We investigated a persistent outbreak of OXA-48 CPE (primarily *Citrobacter freundii*) in a haematological ward of a French teaching hospital by epidemiological, microbiological and environmental methods. Between January 2016 and June 2019, we detected 37 new OXA-48 CPE-colonised and/or ‑infected patients in the haematological ward. In October 2017, a unit dedicated to CPE-colonised and/or ‑infected patients was created. Eleven additional sporadic acquisitions were identified after this date without any obvious epidemiological link between patients, except in one case. Environmental investigations of the haematological ward (June–August 2018) identified seven of 74 toilets and one of 39 drains positive for OXA-48 CPE (seven *C. freundii*, one *Enterobacter sakazakii*, one *Escherichia coli*). Whole genome comparisons identified a clonal dissemination of OXA-48-producing *C. freundii* from the hospital environment to patients. In addition to strict routine infection control measures, an intensive cleaning programme was performed (descaling and bleaching) and all toilet bowls and tanks were changed. These additional measures helped to contain the outbreak. This study highlights that toilets can be a possible source of transmission of OXA-48 CPE.

## Background

Carbapenems represent a last resort antibiotic therapy for patients infected with extended-spectrum β-lactamase-producing Enterobacterales. Thus, the growing prevalence of carbapenemase-producing Enterobacterales (CPE) is of great concern since carbapenemase production is associated with an increased mortality rate [1]. Since the beginning of the 2000s, outbreaks of CPE are reported worldwide and CPE has become endemic in some countries [2]. In 2018, data reported to the European Antimicrobial Resistance Surveillance Network (EARS-Net) concerning invasive isolates indicated that 0.1% and 7.5% of *Escherichia coli* and *Klebsiella pneumoniae,* respectively, were resistant to carbapenem [3]. In France, the number of cases (infections and colonisations) and outbreaks of CPE has steadily increased since 2009. Although most cases are patients with a recent history of travelling or hospitalisation abroad, the number of autochthonous cases, often caused by OXA-48 CPE, is on the rise [4,5]. Patient-to-patient cross-transmission is the main spreading mechanism of CPE during nosocomial outbreaks. Environmental reservoirs, such as contaminated sinks, have been reported as sources of such outbreaks and may act to amplify dissemination [6,7]. Transmission may occur from direct or indirect water contact as well as from droplets generated during water use in health care, such as shower and sink cleaning, hand washing or patient cleaning [6].

Between 2016 and 2019, the haematological ward of our hospital experienced a large and protracted outbreak caused by OXA-48 CPE, mostly *Citrobacter freundii*. The outbreak was successfully controlled only after recognising the toilets were the source of transmission. Here, we report the epidemiological and microbiological investigations.

## Methods

### Setting

Saint Antoine hospital is a 685-bed teaching hospital in Paris, France. It includes a haematological ward with 74 single-bed rooms comprising four units, and a haematological day care unit with 34 beds. In 2018, the ward recorded 923 admissions resulting in a total of 21,308 hospitalisation days.

### Case definitions

Cases were defined as colonised or infected patients identified with an OXA-48 CPE while in the haematological ward between January 2016 and June 2019. Colonised patients were defined as patients in whom OXA-48 CPE was identified only on rectal swabs. Infected patients were defined as patients with at least one clinical sample positive for OXA-48 CPE.

Cases were categorised as imported into the haematological ward when CPE was identified within 48 h after admission. Acquired cases were defined as a CPE detection at least 48 h after admission or on admission if the patient was previously identified as a contact patient in the haematological ward.

A contact patient was defined as any patient cared for by the same healthcare team as a CPE case before implementation of contact precautions.

### Microbiological investigations

Weekly routine rectal samples were recovered with pre-moistened swabs. Samples were directly inoculated on ChromID CARBA SMART (bioMérieux, Marcy-l’Etoile, France) screening agar.

Between January 2016 and June 2018, intermittent environmental sampling was performed to identify potential reservoirs of CPE. Overall, 263 samples were collected from the patient environment (n = 118, including 44 tables, 26 toilet seats, 23 bed frames, 10 mattresses, 10 other surfaces and five armchairs), sinks and shower drains (n = 137), and medical devices (n = 8).

From June 2018 to August 2018, all toilet rims in the haematological ward (n = 74), some toilet tanks (n = 6) and a toilet seat as well as some sink and shower drains (n = 39) were screened for CPE.

Environmental samples from dry or damp surfaces were recovered with sterile cotton-tipped swabs. A pre-enrichment, performed in a tryptic soy broth, was incubated at 37 °C in an aerobic atmosphere for 24 h before plating on ChromID CARBA SMART agar.

Cultured isolates were identified using the MALDI Biotyper based on matrix-assisted laser desorption/ionisation time-of-flight mass spectrometry (MALDI-TOF MS, Bruker, Wissembourg, France).

Antimicrobial susceptibility testing was performed by disk diffusion method on Mueller-Hinton agar (Bio-Rad, Marnes-la-Coquette, France) and interpreted as recommended by the European Committee on Antimicrobial Susceptibility Testing (EUCAST) [8].

When CPE was suspected, the identification of carbapenemase type was confirmed by PCR (Xpert Carba-R assay, Cepheid, Maurens-Scopont, France) or by immunochromatographic assay (RESIST-4 O.K.N.V, Coris BioConcept, Gembloux, Belgium).

OXA-48 CPE isolates from patients and from the environment were sent to the associated National Reference Centre (NRC) for antimicrobial resistance for confirmation and typing. In the NRC, carbapenemase production was reassessed using both the RAPIDEC® CARBA NP test (bioMérieux, Marcy-l’Etoile, France) and another immunochromatographic assay (NG-CARBA 5, NG Biotech, Guipry, France), as previously described [9,10].

### Whole genome sequencing

To distinguish carbapenemase producers belonging to the same sequence type, whole genome sequencing was performed using Illumina technology (Illumina, Evry, France) as previously described [11]. Total DNA was extracted from colonies using the Ultraclean Microbial DNA Isolation Kit (MO BIO Laboratories, Ozyme, Saint-Quentin, France) following the manufacturer’s instructions. De novo assembly and read mappings were performed using CLC Genomics Workbench v10.1 (Qiagen, Les Ulis, France). The acquired antimicrobial resistance genes were identified using Resfinder server v3.1 (https://cge.cbs.dtu.dk/services/ResFinder/) and CARD database (https://card.mcmaster.ca). Phylogeny was performed using CSIphylogeny v1.4 server (https://cge.cbs.dtu.dk/services/CSIPhylogeny/) and visualised using FigTree software v1.4.3 (http://tree.bio.ed.ac.uk/).

### Nucleotide sequence accession numbers

Sequencing reads from the 29 sequenced OXA-48-producing *C. freundii* isolates of sequence type (ST)-22 that have been used to construct the phylogenetic tree have been deposited in the GenBank database under the BioProject accession number PRJNA664303 in the NCBI BioProject database (https://www.ncbi.nlm.nih.gov/bioproject/).

### Infection control measures

During the outbreak (January 2016–June 2019), contact precautions were applied for all cases and contact patients according to French guidelines [12,13]. CPE patients were cohorted inside the haematological ward with dedicated staff.

Since April 2007, active surveillance for CPE colonisation has been performed on admission and thereafter weekly for all patients admitted to the haematological ward. The infection control team implemented a programme to limit in-hospital transmission, including a mandatory training course for all healthcare workers on infection control measures, opportunities for hand hygiene with alcohol hand gels, and contact precautions. Furthermore, hand hygiene and excreta management were evaluated in the ward. Weekly meetings between the infection control team and haematological staff were implemented to help strengthen compliance with the control measures.

Spatio-temporal links were found for CPE-positive patients between January 2016 and September 2017, suggesting a possible cross-transmission via the healthcare workers’ hands despite infection control measures. Consequently, in October 2017, a dedicated six-bed unit for all CPE patients was created with dedicated nurses and nurse assistants, in order to separate CPE-positive patients from other patients hospitalised in the haematological ward.

### Matched case–control study

Variables associated with OXA-48 CPE acquisition were investigated in a matched case–control study. Cases were patients who acquired OXA-48 CPE between January 2017 and June 2018 in the haematological ward. Controls were contact patients of acquired cases. Each case was matched with three control patients according to the exposure time in the unit. Matched controls were randomly selected. Collected data included age, sex, haematopoietic stem cell transplantation, antibiotic exposure and immunosuppressive therapy (defined as treatment with chemotherapy, immunosuppressive drugs, radiotherapy or corticosteroids) in the previous month, room number and length of stay in the previous 6 months in our hospital. Univariate analyses were performed using conditional logistic regression. Statistical analysis was performed using Stata software (v15.1, StataCorp, College Station, Texas, United States).

## Results

### Case characteristics

**Figure 1:**
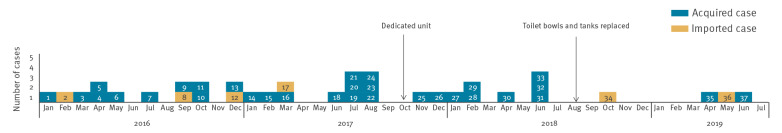
Epidemiological curve of cases with OXA-48-producing Enterobacterales infection or colonisation in the haematological ward, France, January 2016–­June 2019 (n = 37)­

Between January 2016 and June 2019, 37 new OXA-48 CPE cases were detected in the haematological ward (Figure 1 and Supplementary Table S1). Of these, 31 were considered as acquired cases, and six were identified as imported cases. The median age was 59.5 years (interquartile range: 45.0–69.4) and 19 patients were male. Among the 37 OXA-48 CPE cases, 32 were initially detected by rectal screening, and 21 developed an infection. These included 13 urinary tract, seven bloodstream, and one pneumonia infection. Three patients (Cases 11, 22 and 34) carried a strain harbouring both *bla*_OXA-48_ and *bla*_NDM_ genes, and two of these cases were acquired (Cases 11 and 22).

Cases were categorised as imported into the haematological ward when CPE was identified within 48 h after admission. Acquired cases were defined as a CPE detection at least 48 h after admission or on admission if the patient was previously identified as a contact patient in the haematological ward.

A total of 78 OXA-48 CPE were detected including 22 *C. freundii*, 19 *E. coli*, 15 *K. pneumoniae*, seven *Klebsiella oxytoca*, six *Enterobacter cloacae*, two *Citrobacter koseri*, two *Enterobacter aerogenes*, one *Hafnia alvei*, one *Kluyvera cryocrescens*, one *Citrobacter amalonaticus*, one *Morganella morganii*, and one *Raoultella ornithinolytica*. Eighteen patients harboured at least two different CPE (Supplementary Table S1).

In addition, two NDM-CPE-positive patients were hospitalised in the haematological ward (one imported in 2017 and one acquired in 2019).

**Figure 2:**
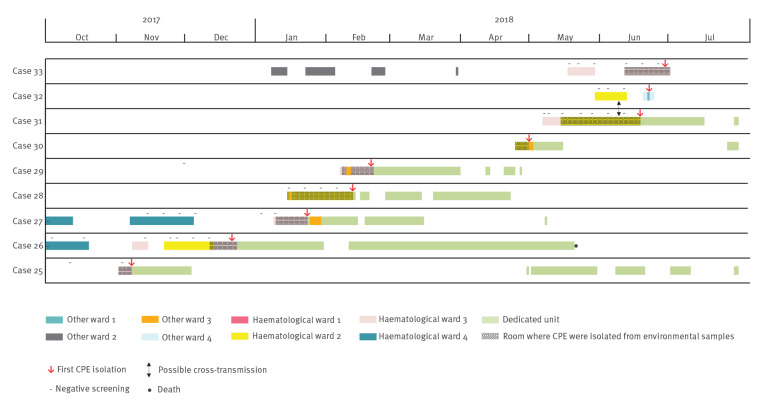
Timeline of the detection of OXA-48-producing Enterobacterales cases, France, November 2017–July 2018 (n = 9)

In October 2017, a dedicated unit was opened for CPE-positive patients, but this did not help to contain the outbreak; 13 new cases were identified in the following months up until the study endpoint of June 2019. Of these, 11 cases were acquired, and nine occurred between November 2017 and June 2018 (Figure 2), suggesting the presence of a potential environmental reservoir of CPE. Only one (Case 32) was identified as a contact of a known case.

CPE: carbapenemase-producing Enterobacterales.

Coloured rectangles represent the presence of the patient in the corresponding ward. Red arrows represent the detection of a CPE in a screening or clinical sample. Minus signs represent a negative CPE screening test. Double arrows represent a possible cross-transmission. The haematological ward consists of four units represented by different colours in the figure (numbered 1 to 4) and a dedicated unit. Other wards are unrelated to the haematological ward.

### Environmental investigations

Between January 2016 and June 2018, all 263 environmental samples were negative for CPE. From June 2018 to August 2018, eight samples of 119 were positive for OXA-48 CPE in two units including seven of 74 toilet rims and one of 39 sink drains. Strains isolated by culture included *C. freundii* (n = 7), *Enterobacter sakazakii* (n = 1) and *E. coli* (n = 1). Of the seven *C. freundii* isolates, five belonged to ST-22, one to ST-253-like, and one was not typed. In contrast, 104 toilets randomly selected elsewhere within the hospital were screened and only two were *C. freundii* OXA-48-positive (p = 0.034).

### Comparison of OXA-48-producing *Citrobacter freundii* isolates

Sequence type was determined for 34 CPE isolates. We focused our attention on *C. freundii* since all acquired isolates (n = 17) belonged to the same sequence type (ST-22). In contrast, the remaining OXA-48 CPE species (nine *E. coli*, four *K. pneumoniae*, two *K. oxytoca*, one *E. cloacae*, one *C. freundii*) belonged to different STs (Supplementary Table S1).

**Figure 3:**
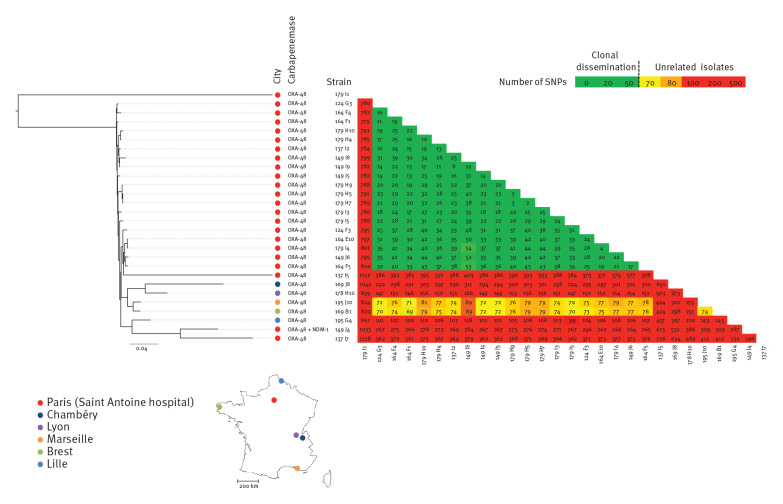
Phylogenetic tree of OXA-48-producing *Citrobacter freundii* sequence type (ST)-22 isolates, France, 2016–2019 (n = 28)

SNP: single nucleotide polymorphism; NDM: New Delhi metallo-beta-lactamase.

We compared 23 clinical and six environmental (toilet) isolates of OXA-48-producing *C. freundii* by whole genome sequencing (Figure 3). Twenty-two were identified in the haematological ward (17 clinical and five environmental strains (179 H9, 179 H10, 179 I1, 179 I3 and 179 I4)), two were identified elsewhere in our hospital (one clinical strain (137 I7) and one environmental strain (179 I5)) and five unrelated strains came from the collection of the NRC (169 J8, 178 H10, 195 J10, 169 B1 and 195 G4).

The map of France shows the location of CPE isolates. The scale bar under the phylogenetic tree indicates the number of SNPs per position of common sequences.

All 24 OXA-48-producing *C. freundii* identified in our hospital belonged to ST-22, except Case 8, which was an imported case. In addition, determination of single nucleotide polymorphisms (SNPs) in the whole genome revealed that all ST-22 OXA-48-producing *C. freundii* belonged to the same cluster (< 50 SNPs between strains) except for five isolates: 179 I1 (environmental strain from haematology), 137 I7 (patient from another ward in the same hospital), 149 J4 (Case 22, patient positive for both OXA-48 and NDM-1), 137 I5 (Case 13), and 244 E5 (Case 37). Three patients (Cases 22, 13 and 37) presented no traditional risk factors for CPE carriage (i.e. previous travel or hospitalisation abroad).

### Matched case–control study

Nineteen acquired CPE cases identified between January 2017 and June 2018 (Cases 14 to 16 and 18 to 33) were matched to 57 controls. The only factor significantly associated with CPE acquisition was hospitalisation in a room with a toilet that was positive for OXA-48 CPE (odds ratio = 6.2; 95% confidence interval: 2.0–19.6; p = 0.002) (Table).

**Table:** Matched case–control study of a CPE outbreak in a haematological ward, France, January 2017–June 2018 (n = 76)

Variable	Cases (n = 19)	Controls (n = 57)	OR	95% CI	p
Median (IQR)					
Age in years	58 (40–70)	63 (55–67)	1.0	0.9–1.0	0.38
Length of stay (previous 6 months, in days )	28 (13–71)	54 (27–96)	1.0	1.0–1.0	0.11
n (%)					
Men	8 (42)	37 (65)	0.4	0.1–1.2	0.10
Haematopoietic stem cell transplantation	14 (74)	39 (68)	1.3	0.4–4.3	0.65
Antibiotic exposure (previous month)	18 (95)	45 (80)	4.5	0.5–36.6	0.17
Immunosuppressive therapy (previous month)	17 (89)	51 (89)	1.0	0.2–5.3	1.00
Hospitalisation in a room with OXA-48 CPE-positive toilet	14 (74)	15 (26)	**6.2**	**2.0–19.6**	**0.002**

CI: confidence interval; CPE: carbapenemase-producing Enterobacterales; IQR: interquartile range; OR: odds ratio.

Multiple variables can apply to more than one case.

### Outbreak control measures

Following the identification of the toilets as a potential source of the outbreak, intensive toilet cleaning with descaling and bleaching (initially daily, then weekly) was implemented. Afterwards, 23 environmental samples were taken (including 21 toilet rims and two drains), and only one toilet remained positive for OXA-48-producing *C. freundii*. This toilet was successfully re-decontaminated by performing a single additional cleaning and bleaching. In August 2018, all toilets bowls and tanks in two units with environmental CPE-positive samples were replaced by rimless toilets. Rimless toilets are easier to clean and reduce the risk of limescale deposits. After implementation of the environmental measures, the incidence of new CPE cases declined, and only two unrelated CPE cases (Cases 35 and 37) (Figure 1 and Supplementary Table S1) were acquired 8 and 10 months, respectively, after this measure. The origin of these two CPE cases remain undetermined.

## Discussion

We report here the persistent transmission of OXA-48 CPE in a haematological ward and provide arguments to support the role of toilets in the transmission of CPE. Firstly, environmental sampling confirmed that toilets were contaminated by CPE and represented a potential reservoir. This could explain the persistence of the transmission despite the implementation of infection control measures including a cohorting unit dedicated to CPE cases. Secondly, the only factor significantly associated with CPE acquisition was hospitalisation in a room with OXA-48-positive toilets. Thirdly, the incidence of CPE cases declined after intensive toilet cleaning, and subsequent replacement with rimless toilets. Through ongoing surveillance, we reported only two new unrelated acquired CPE cases, 8 and 10 months apart respectively, after this measure.

Patient-to-patient transmission via the hands of healthcare workers has been considered as the major route of CPE transmission [14,15]. However, the water environment is widely reported as a reservoir for hospital-acquired transmission of carbapenem-resistant organisms. Drains, sinks and faucets are the most frequently contaminated sites, with *Pseudomonas aeruginosa* as the predominant microorganism [6,7,16]. A combination of interventions including reinforcement of infection control measures, environmental cleaning and replacement of all the toilets has been shown to be successful in controlling the outbreaks [17,18]. However, to our knowledge, toilets have never been shown to be exclusively responsible for CPE transmission.

When flushed, toilets splash and produce droplet aerosols. Aerosolisation of microorganisms from contaminated toilets during flushing has repeatedly been demonstrated for various toilet types and organisms for the past 50 years [19]. Transmission of microorganisms from toilets is attributed to splashing directly on the patient or by contamination of the environment. Recently, Buchan et al. found a high prevalence of carbapenemase-producing bacteria in sink drains, especially next to toilets, suggesting a contamination of the sink via droplets during toilet flushing [20].

If toilets are indeed a reservoir and a potential source of CPE transmission, then additional interventions such as optimisation of toilet disinfection protocols (such as serial cleaning with descaling and bleaching), rimless toilet bowls, patient hand hygiene reinforcement, use of removable seats to optimise disinfection, or patient education to close the toilet lid before flushing, should be implemented.

In our cohort, 21 of the total 37 patients had a CPE infection. Of those infections, seven were bacteraemia, including one case caused by *C. freundii*. This proportion is considerably higher than that reported in the literature [21]. This high proportion of infected patients is probably due to the fact that most of the patients were immunocompromised. Because our outbreak involved multiple Enterobacterales species, we hypothesised that the *bla*_OXA-48_‑carrying plasmids spread among various species in the same patient, as described by Conlan et al. [22]. Indeed, the high conjugation frequency of the prototypical *bla*_OXA-48_‑carrying plasmid has been attributed to the disruption of the *tir* gene, encoding a transfer inhibition protein, by insertion of the transposon Tn*1999* where *bla*_OXA-48_ is localised [23]. The fact that, on one hand, all OXA-48-producing *C. freundii* isolates were clonal and, on the other hand, the OXA-48 CPE species other than *C. freundii* belonged to different STs, suggests that a transmission of *C. freundii* occurred first, followed by a transfer of the OXA plasmid to other Enterobacterales present in each patient’s gut. However, the reason why the *C. freundii* species (especially the ST-22 cluster), rather than *K. pneumoniae* or *E. cloacae*, played a key role in the dissemination of OXA-48 via toilets as a reservoir needs further investigation.

Our study has several limitations. Firstly, as a single centre study, its generalisation is limited. Secondly, there was no assessment of adherence to the infection control team recommendations. Thirdly, as with any observational study, other events (i.e. antibiotic use, alcohol hand rub use) may have had an impact on the reduction of CPE acquisition. Finally, the infection control strategies that were implemented included bundled approaches (reinforcement of hand hygiene, cleaning with descaling, bleaching and replacing the standard toilets with rimless toilets) making it difficult to demonstrate the relative effect of each measure individually.

## Conclusion

This outbreak highlights the possible role of toilets as a source of transmission of OXA-48 CPE. It was successfully controlled only after replacing all the toilets in the ward. When confronted with a protracted outbreak, infection control teams should take into account uncommon environmental reservoirs in their investigation and enactment of control measures.

## Supplementary Data

156000 Supplementary Table S1
