# Supplementary material for: Outbreak of OXA-48-producing Enterobacterales in a haematological ward associated with an uncommon environmental reservoir, France, 2016 to 2019
Source: Euro Surveill. 2021 May 27;26(21):2000118. doi: 10.2807/1560-7917.ES.2021.26.21.2000118 (PMC8161731; doi:10.2807/1560-7917.ES.2021.26.21.2000118)
Supplement: Supplementary Table S1 [file 2000118_Jolivet_SupTabS1.pdf]

Supplementary Table S1. Characteristics of OXA-48-producing Enterobacterales cases in the haematological ward, France, January 2016 - June 2019 (n=37)

Case number	Acquired or imported case	Date	Species	ST	Isolate reference	Carbapenemase
1	Acquired	Jan-16	*K. pneumoniae*	ND		OXA-48
2	Imported	Feb-16	*E. coli*	ST-2973		OXA-48
		Feb-16	*E. aerogenes*	ND		OXA-48
3	Acquired	Mar-16	*C. freundii*	ND		OXA-48
		Mar-16	*K. pneumoniae*	Unknown		OXA-48
4	Acquired	Apr-16	*E. aerogenes*	ND		OXA-48
5	Acquired	Apr-16	*K. pneumoniae*	ST-307		OXA-48
6	Acquired	May-16	*C. freundii*	ST-22	124 G3	OXA-48
7	Acquired	Jul-16	*C. freundii*	ST-22	124 F3	OXA-48
		Jul-16	*E. coli*	ST-349		OXA-48
		Jul-16	*K. oxytoca*	ND		OXA-48
		Jul-16	*E. cloacae*	ND		OXA-48
8	Imported	Sep-16	*C. freundii*	ST-8		OXA-48
		Sep-16	*E. cloacae*	ND		OXA-48
		Sep-16	*K. pneumoniae*	ND		OXA-48
9	Acquired	Sep-16	*E. coli*	ST-636		OXA-48
10	Acquired	Oct-16	*C. koseri*	ND		OXA-48
		Oct-16	*C. freundii*	ND		OXA-48
		Nov-16	*K. pneumoniae*	ST-2459		OXA-48
		Dec-16	*E. coli*	ST-828		OXA-48
		Mar-17	*C. amalonaticus*	ND		OXA-48
		Apr-17	*M. morganii*	ND		OXA-48
11	Acquired	Oct-16	*E. coli*	ST-410		OXA-48, NDM-5
12	Imported	Dec-16	*E. coli*	ST-88		OXA-48
13	Acquired	Dec-16	*C. freundii*	ST-22	137 I5	OXA-48
14	Acquired	Jan-17	*C. freundii*	ND		OXA-48
		Jan-17	*K. pneumoniae*	ND		OXA-48
15	Acquired	Feb-17	*E. coli*	ST-88		OXA-48
		Mar-17	*C. freundii*	ST-22	137 I2	OXA-48
		Mar-17	*E. cloacae*	ST-114		OXA-48
16	Acquired	Mar-17	*E. coli*	ST-46		OXA-48
17	Imported	Mar-17	*E. coli*	ST-354		OXA-48
		May-17	*K. oxytoca*	ST-43		OXA-48
18	Acquired	Jun-17	*C. freundii*	ST-22	149 I8	OXA-48
19	Acquired	Jul-17	*C. freundii*	ST-22	149 I9	OXA-48
20	Acquired	Jul-17	*K. pneumoniae*	ST-16		OXA-48
21	Acquired	Jul-17	*C. freundii*	ND		OXA-48
		Jul-17	*K. pneumoniae*	ND		OXA-48
		Oct-17	*H. alvei*	ND		OXA-48
		Oct-17	*E. coli*	ND		OXA-48
22	Acquired	Aug-17	*C. freundii*	ST-22	149 J4	OXA-48, NDM-1

		Aug-17	*E. coli*	ND		OXA-48
		Aug-17	*K. pneumoniae*	ND		NDM
		Sep-17	*K. oxytoca*	ST-144 SLV		OXA-48
23	Acquired	Aug-17	*C. freundii*	ST-22	149 J6	OXA-48
24	Acquired	Aug-17	*C. freundii*	ST-22	149 J5	OXA-48
		Aug-17	*R. ornithinolytica*	ND		OXA-48
		Aug-17	*E. coli*	ND		OXA-48
		Sep-17	*K. oxytoca*	ND		OXA-48
		Apr-18	*K. pneumoniae*	ND		OXA-48
25	Acquired	Nov-17	*K. pneumoniae*	ND		OXA-48
26	Acquired	Dec-17	*C. freundii*	ST-22	164 E10	OXA-48
		Dec-17	*K. oxytoca*	ND		OXA-48
		Jan-18	*E. cloacae*	ND		OXA-48
27	Acquired	Jan-18	*C. freundii*	ST-22	164 F1	OXA-48
		Jan-18	*C. koseri*	ND		OXA-48
28	Acquired	Feb-18	*C. freundii*	ST-22	164 F5	OXA-48
		Feb-18	*K. pneumoniae*	ND		OXA-48
		Mar-18	*E. coli*	ND		OXA-48
29	Acquired	Feb-18	*C. freundii*	ST-22	164 F4	OXA-48
		Jun-18	*E. coli*	ND		OXA-48
		Jun-18	*K. oxytoca*	ND		OXA-48
		Jul-18	*K. pneumoniae*	ND		OXA-48
30	Acquired	Apr-18	*C. freundii*	ST-22	179 H4	OXA-48
31	Acquired	Jun-18	*C. freundii*	ST-22	179 H5	OXA-48
		Jul-18	*K. pneumoniae*	ND		OXA-48
		Jul-18	*E. coli*	ND		OXA-48
		Oct-18	*K. oxytoca*	ND		OXA-48
		Nov-18	*K. cryocrescens*	ND		OXA-48
		Jan-19	*E. cloacae*	ND		OXA-48
32	Acquired	Jun-18	*E. cloacae*	ND		OXA-48
		Sep-18	*E. coli*	ND		OXA-48
33	Acquired	Jun-18	*C. freundii*	ST-22	179 H7	OXA-48
34	Imported	Oct-18	*E. coli*	ND		OXA-48, NDM
35	Acquired	Apr-19	*E. coli*	ND		OXA-48
36	Imported	May-19	*E. coli*	ND		OXA-48
37	Acquired	Jun-19	*C. freundii*	ST-22	244 E5	OXA-48
		Jun-19	*K. pneumoniae*	ND		OXA-48

ND: not done; ST: sequence type
C. freundii ST-22 appears in red

This supplementary material is hosted by *Eurosurveillance* as supporting information alongside the article "Outbreak of OXA-48-producing Enterobacterales in a haematological ward associated with an uncommon environmental reservoir, France, 2016-2019", on behalf of the authors, who remain responsible for the accuracy and appropriateness of the content. The same standards for ethics, copyright, attributions and permissions as for the article apply. Supplements are not edited by *Eurosurveillance* and the journal is not responsible for the maintenance of any links or email addresses provided therein."
